# tRNA derived fragment *(tRF)-3009* participates in modulation of IFN-α-induced CD4^+^ T cell oxidative phosphorylation in lupus patients

**DOI:** 10.1186/s12967-021-02967-3

**Published:** 2021-07-13

**Authors:** Guannan Geng, Huijing Wang, Weiwei Xin, Zhe Liu, Jie Chen, Zhang Danting, Fei Han, Shuang Ye

**Affiliations:** 1 Department of Rheumatology, Renji Hospital, Shanghai Jiaotong University School of Medicine, Shanghai, China; 2 Center for Excellence in Brain Science and Intelligence Technology, Chinese Academy of Sciences, Shanghai, China; 3 Key Laboratory of Kidney Disease Prevention and Control Technology, Kidney Disease Center, The First Affiliated Hospital, Zhejiang University School of Medicine, Institute of Nephrology, Zhejiang University, Hangzhou, China; 4 Department of Orthopaedics, Renji Hospital, Shanghai Jiaotong University School of Medicine, Shanghai, China

**Keywords:** tRNA derived fragments (tRFs), Systemic lupus erythematosus (SLE), CD4^+^ T cells, Oxidative phosphorylation (OXPHOS), Type I interferon (IFN)

## Abstract

**Background:**

Accumulating evidence suggests tRNA-derived fragments (tRFs) play important roles in cellular homeostasis. Here we aimed to explore aberrant expression of tRFs in CD4^+^ T cells from patients with systemic lupus erythematosus (SLE) and their potential function in the SLE pathogenesis.

**Methods:**

First, small RNA sequencing was performed on CD4^+^ T cells from four SLE patients and three healthy controls (HCs). Candidate tRFs were then validated in CD4^+^ T cells from 97 SLE patients and their relevant disease controls using qRT-PCR. Then sequencing was used to investigate the profiles of HC-derived CD4^+^ T cells transfected with *tRF-3009*. Lastly, *tRF-3009* siRNA or *tRF-3009* mimics were transfected into CD4^+^ T cells with/without IFN-α. Changes in oxygen consumption rate (OCR), ATP, and ROS production were analyzed.

**Results:**

We identified 482 differentially expressed tRFs from SLE CD4^+^ T cells and chose tRF-3009 for further analysis due to its upregulation and the positive correlations between its expression and SLEDAI, active lupus nephritis and serum IFN-α levels. In vitro, *tRF-3009* over-expressing CD4^+^ T cell profiling and putative analysis linked this product to the type I IFN and oxidative phosphorylation (OXPHOS) pathways. Interestingly, IFN-α is capable of inducing ROS and ATP production in CD4^+^ T cells, while knockdown of *tRF-3009* reversed this process. Overexpression of *tRF-3009* in CD4^+^ T cells alone was sufficient to upregulate OCR, ROS, and ATP production.

**Conclusions:**

Our study is the first to link aberrant tRF expression and SLE. *tRF-3009* may participate in metabolic modulation of IFN-α-induced CD4^+^ T cell OXPHOS in lupus.

**Supplementary Information:**

The online version contains supplementary material available at 10.1186/s12967-021-02967-3.

## Introduction

Systemic lupus erythematosus (SLE) is a complex autoimmune disease with a largely undefined pathogenesis [1]. Recently, changes in the metabolism of immune cells have been implicated in SLE [2, 3]. For example, SLE CD4^+^ T cells were identified by their increased glycolysis and mitochondrial oxidative phosphorylation (OXPHOS) [4, 5]. There is a growing belief that metabolic modulators (metformin and/or 2-Deoxy-D-glucose) could restore their cellular function and reverse certain disease phenotypes, this theory is supported by several studies using mouse models of lupus [4, 6, 7]. In addition, our recent clinical trials suggest that the addition of metformin to the standard of care for these patients may reduce the risk of a lupus flare [8].

Transfer RNA-derived fragments (tRFs) are an abundant class of small non-coding RNAs (ncRNAs) that exist in most organisms [9–12]. tRFs can be constitutively produced as a result of various pathophysiologies and their sequence is based on the different cleavage products produced from the mature and precursor tRNA transcripts [13]. Growing evidence suggests that tRFs, like other ncRNAs, can serve as active modulators of cellular homeostasis [14, 15]. Recently, tRFs have been implicated in numerous diseases, including multiple cancers, neurodegenerative disorders, viral infections, and metabolic disorders. Accumulating evidence have shown their biomarker potential and determined the molecular mechanisms of tRFs‐mediated pathological processes [15, 16]. Although their biological functions are still poorly understood, recent studies have shown sperm tRNA fragments might affect human metabolic disorders [17, 18], indicating that tRFs may participate in metabolism process.

Surprisingly, we have found tRFs were abnormally expressed in CD4^+^ T cells isolated from SLE patients. To the best of our knowledge, tRFs have never been studied in SLE. This study aims to explore the potential effects of candidate tRFs in immunometabolism on the pathogenesis of SLE.

## Materials and methods

### Patients and study design

This study included 187 participants and their clinical data are listed in Additional file 6: Table S1. Four new-onset SLE patients and three healthy controls (HCs) were chosen randomly using in the primary small RNA profiling study and the validation cohort consisted of 97 SLE patients , and 25 disease controls [10 rheumatoid arthritis (RA) patients and 15 ankylosing spondylitis (AS) patients] and 20 HCs. An additional 38 SLE patients were enrolled for further IFN-α-related assay. All patients fulfilled the classification criteria for SLE [19], RA [20], or AS [21], respectively, and the enrollment was completed at the Department of Rheumatology, Renji Hospital, Shanghai Jiaotong University School of Medicine, from June 2017 to December of 2018. The SLE disease activity index (SLEDAI) [22] was assessed and all patients with  >  500 mg/24 h proteinuria were defined as experiencing active lupus nephritis at the time of enrollment. Among all enrolled SLE patients, 71 subjects with stable disease activity, 31 with moderate disease activity and 37 with severe disease activity. The SLEDAI score in all SLE patients was 5.77  ±  4.96. And 68 SLE patients were experiencing active disease. Based on the prednisone dose that patients taking, SLE patients were divided into low/median/high dose (<  20 mg/d or  ≤  20 mg/d and  <  50 mg/d or  ≤  50 mg/d) group. Any subjects with acute or chronic infections were excluded and the study protocol was reviewed and approved by the institutional ethics committee, and written informed consent was obtained from all participants.

### Isolation of CD4^+^ T cells

The peripheral blood lymphocytes (PBMCs) were isolated from HCs and SLE patients using Ficoll gradient centrifugation within 6 h of collection of the samples. CD4^+^ T cells were then purified after immunomagnetic separation with the human CD4^+^ T cell isolation kit (BD Biosciences) according to the manufacturer’s instructions. The isolated human primary CD4^+^ T cells were lysed in Trizol Reagents (Sigma) or used to perform in vitro transfection. Samples from the validation cohort were used for qRT-PCR.

### Cell culture and transfection

CD4^+^ T cells from HCs were cultured in Roswell Park Memorial Institute (RPMI)-1640 medium (Hyclone) supplemented with 10% fetal bovine serum (FBS) and 100 U/mL IL-2 at 37 °C in a humidified atmosphere containing 5% CO_2_ (Thermo Fisher). For transfection, CD4^+^ T cells were seeded at a density of 0.5 × 10^6^ cells/well and three types of *tRF-3009* or three types of negative control (single-stranded RNA, ssRNA) (Guangzhou RiboBio Co., Ltd.) were transfected using RNAimax (Thermo Fisher) according to the manufacturer’s protocols. After 48 h of incubation at 37 °C, cells were harvested for small RNA sequencing.

To evaluate the metabolic changes CD4^+^ T cells were seeded at a density of 1  ×  10^6^ cells/well and stimulated with/without 1  ×  10^5^ U/mL IFN-α and transfected with or without 20 µM *tRF-3009* siRNA or 20 µM *tRF-3009* mimic for 24 h. Metformin (Met) or 2-Deoxy-D-glucose (2-DG) were added at 1 mM for 24 h, and phytohemagglutinin (PHA) was added at 1 µg/mL as indicated.

### Deep sequencing

Total RNA was extracted from the primary human CD4^+^ T cells using the TRIzol Reagent (Invitrogen) and the quality of the RNA samples was evaluated by using a Nanodrop 2000c Spectrophotometer (Thermo Fisher Scientific Inc.) and then size-fractionated on a 15% polyacrylamide gel electrophoresis (PAGE) gel to collect the 18–60nt fraction. The 5′ and 3′ RNA adapters were ligated to the RNA pool, and then this was subjected to RT-PCR to generate the sequencing libraries. PCR products were purified and then sequenced using a HiSeq 2000 sequencing system (BGI Tech). tRFs were aligned to the tRFdb (http://genome.bioch.virginia.edu/trfdb) and the transcripts were trimmed and aligned to the human genome (hg19). Differentially expressed tRFs and the transcripts were filtered using the following criteria: |Fold change  ≥  2.0| and P value  <  0.05 and |Fold change  ≥  1.5| and FDR  <  0.05, respectively.

### qRT-PCR validation of candidate tRFs and other mRNAs

Quantification of tRFs was completed using the same methods as those for analyzing PIWI-interacting RNAs (piRNAs) with some modifications [23]. Isolated total tRFs RNA or mRNA (1.0 μg) was polyadenylated at 37 °C for 20 min with poly(A) polymerase (NEB) and reverse transcription was performed using the Superscript II Reverse Transcriptase kit (Takara). RNA transcript levels were measured by qRT-PCR using SYBR Green PCR Master Mix. After adding forward and reverse primers, the mixtures were incubated at 95 °C for 10 min, followed by 40 cycles of 95 °C for 10 s and 58 °C for 10 s. All experiments were performed in biological triplicate and the data were analyzed using the comparative cycle threshold (Ct) method. U6/β-actin was used as an internal control and the relative quantification was calculated using the following equation: Amount of target gene = 2^−ΔCt^ where ΔCt = Ct _target genes_  −  Ct _U6/β-actin_. Gene-specific primers are listed in Additional file 7: Table S2. All samples were performed in triplicate.

### Serum IFN-α level in lupus patients

Serum was obtained from SLE patients and serum IFN-α levels were measured using human IFN alpha ELISA kits (Raybiotech, lnc) according to the manufacturer’s instructions [24].

### Bioinformatics analysis

The functions of the differentially expressed tRF target genes were investigated using Gene Ontology (GO) annotation and Kyoto Encyclopedia of Genes and Genome (KEGG) pathway analysis. Hierarchical clustering of the differentially expressed genes (DEGs) according to their biological process (BP), cellular component (CC) and molecular function (MF) categories were completed using GO analysis and used to elucidate genetic regulatory networks (http://www.geneontology.org). Pathway analysis using graphical diagrams was performed to explore the DEG pathways using the Pathway database (http://www.genome.jp/kegg/) and a co-expression network was built using the Cytoscape software (version 3.6.1). Significance was determined by P value and FDR. And the protein–protein interaction network (PPI) of the tRF-3009 interacting proteins was constructed and the module analysis was performed using STRING database.

The target binding sites of the tRFs (targeting both the 3′UTR region and promoter regions of all annotated genes) were predicted using the miRanda database (http://www.microrna.org/microrna/home.do). Significant 3′UTR regions and promoter regions were identified using the following criteria: energy ≤  − 15 and score  ≥  140 and energy ≤  − 20 and score ≥ 150, respectively.

### Measurements of metabolic changes

Oxygen consumption rate (OCR) was measured using an XF96 Extracellular Flux Analyzer under mitochondrial stress test conditions (Seahorse). Assay buffer was prepared from non-buffered RPMI medium (Sigma) supplemented with 10 mM glucose, 2 mM glutamine, and 1 mM pyruvate. Baseline OCR values were averaged between technical replicates for the first three successive time intervals. Extracellular lactate production was measured using a Glucose-lactic acid analyzer (SBA-40E). Mitochondrial membrane potential (△Ψm) was assessed using 5,5,6,6′-tetrachloro-1,1,3,3′-tetraethyl benzimidazolyl carbocyanine iodide (JC-1, green^+^ red^−^ cells%, Sigma). JC-1 is an ideal fluorescent probe whose mitochondrial uptake is directly related to the magnitude of the mitochondrial membrane potential [25]. The greater the mitochondrial uptake, the greater concentration of JC-1 aggregate forms which have a red fluorescent emission signal, as opposed to the JC-1 monomer that fluoresces green [25, 26]. The ratio of red to green fluorescence is often applied to measure the ratio of mitochondrial depolarization. Intracellular ATP was measured using the ATP Determination Kit. ROS were measured using DCFH-DA dye (Thermo Fisher), flow cytometry and a Fluorometric Intracellular Ros Kit (Sigma) [27]. Blank medium was used as the negative control, and PHA was used as the positive control. PHA belongs to high molecular glycoprotein family and has the activity of promoting the mitosis of PBMCs [28].

### RNA pull down

The healthy donor-derived CD4^+^ T cells were transfected with 5′-biotinylated synthesized tRF-3009 or a random sequence. CD4^+^  T cells were collected in an extraction buffer 48 h post transfection. After separating the lysate by centrifugation at 14000 rpm for 15 min, the cell extract was incubated with streptavidin-coated microbeads overnight at 4 °C, following the manufacturer’s protocols (μMACS, Miltenyi Biotec.). The proteins interacting with the tRF-3009 were pulled down using magnetic separator, followed by three washes. The proteins were resolved on PAGE, followed by silver staining. Gels fragments that contained bands of the protein of interest were excised and analyzed by mass spectrometry (LC/MS).

### Statistical analysis

Data are presented as the mean  ±  SD. Two-tailed Student’s t test or Mann–Whitney test was used to evaluate the differences between the pairs of groups. Correlations between clinical information and tRF expression were evaluated using Pearson’s correlation test. All statistical analyses were performed using GraphPad Prism (version 6.0) and SPSS software (version 21.0). A P value of  <  0.05 was considered statistically significant.

## Results

### Differentially expressed tRFs in CD4^+^ T cells from SLE patients and HCs

**Fig. 1:**
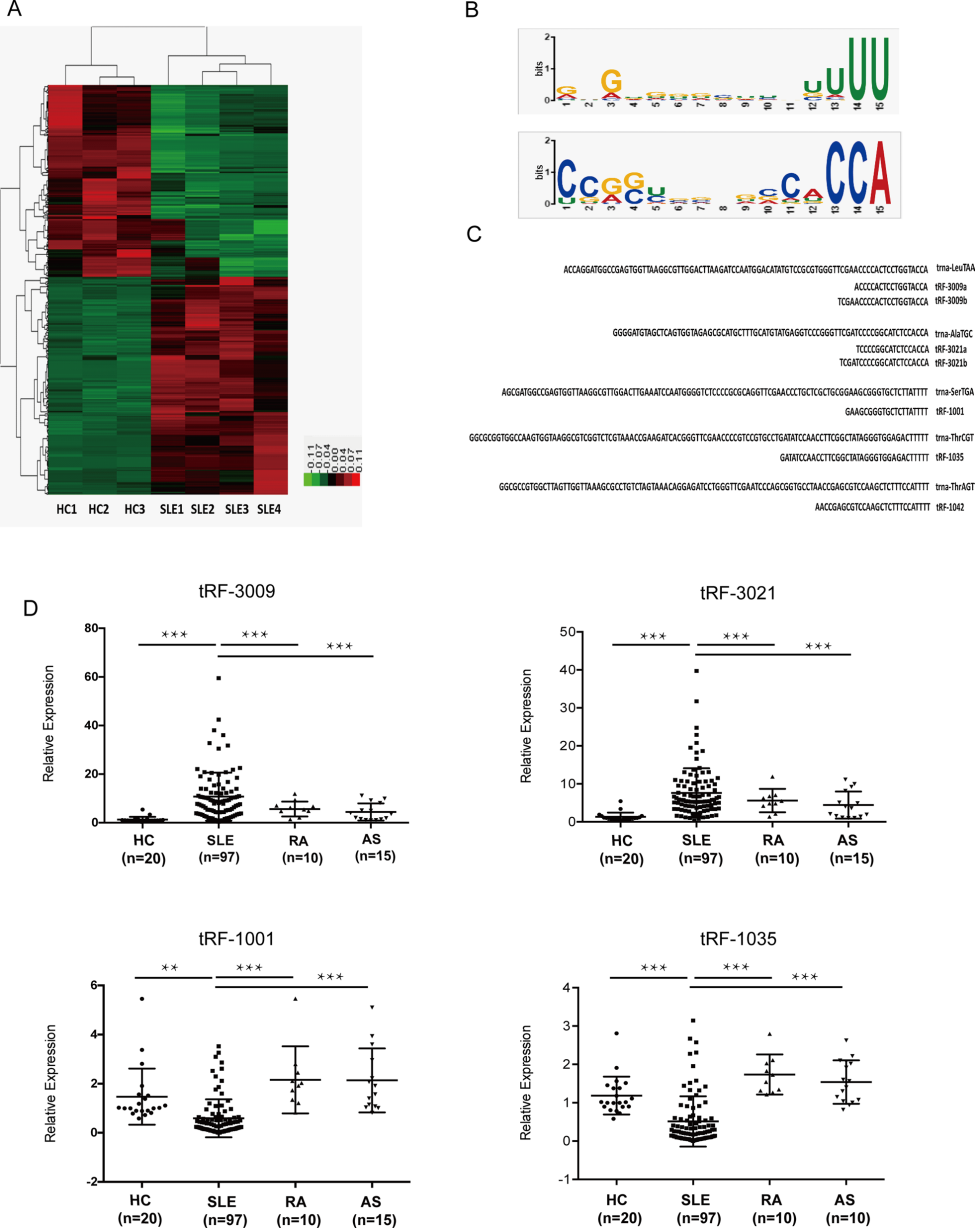
Identification of differentially expressed tRFs in CD4^+^ T cells from systemic lupus erythematosus (SLE) patients and healthy controls (HCs). **A** Heatmap of differentially expressed tRFs between SLE patients (n  =  4) and HCs (n  =  3). The red indicates upregulated genes, and the green indicates downregulated genes. **B** Motif prediction showed the 3′ end sequence characteristics of upregulated tRFs (upper panel) and downregulated tRFs (lower panel). **C** Sequences of the candidate differentially expressed tRFs and their precursors. **D** Expressions of candidate tRFs (tRF-3009, tRF-3021, tRF-1001 and tRF-1035) in CD4^+^ T cells of SLE patients, rheumatoid arthritis (RA) patients, ankylosing spondylitis (AS) patients and HCs. The expression trends of four tRFs was consistent with the small RNA sequencing results. qRT-PCR was performed on RNA samples from 97 SLE patients, 10 RA patients, 15 AS patients and 20 HCs. Data are presented as 2^−ΔCt^ relative to U6 expression. **P  <  0.01; ***P  <  0.001

To determine the differential expression of tRFs in CD4^+^ T cells from SLE patients, small RNA sequencing was performed using cells from 4 SLE patients and 3 healthy controls. In addition to the regular peaks associated with miRNA expression, significantly aberrant expression of tRNA-derived small RNAs was observed in these samples (Fig. 1A). A total of 482 tRFs were significantly differentially expressed in our sequencing data (fold change  ≥  2 or  ≤  0.5 and P  <  0.05), of which 220 tRFs were up-regulated and 262 tRFs were down-regulated. Among the 482 dysregulated tRFs, sequence analysis of the abnormal tRNAs revealed the key enrichment of a common sequence motif: with the majority of the upregulated tRFs derived from the 3′ end of mature tRNAs with a ‘CCA’ tail. While the downregulated tRFs were predominantly produced from the 3′ end of tRNA precursors (Fig. 1B). Based on their sequences, these abnormally expressed small RNAs were derived primarily from tRNA-Leu, followed by tRNA-Ala, tRNA-Ser, and tRNA-Thr (Fig. 1C; Additional file 8: Table S3).

### Identification of the candidate SLE related tRFs from CD4^+^ T Cells

To further confirm the expression levels of the tRFs in SLE patients, the expression levels of the four most significant candidate tRFs were validated by qRT-PCR. Consistent with the tRF-seq data, the expression levels of *tRF-3009* and *tRF-3021* were both significantly higher in SLE samples compared with HCs and disease controls (AS or RA patients; P  <  0.001), while the *tRF-1001* and *tRF-1035* expression levels were significantly lower in these samples (P  <  0.01; Fig. 1D). In addition, these tRFs expressions were not significantly different between the disease control groups and HC group.

### tRF-3009 expression correlates with disease activity index, lupus nephritis and serum IFN-α levels

**Fig. 2:**
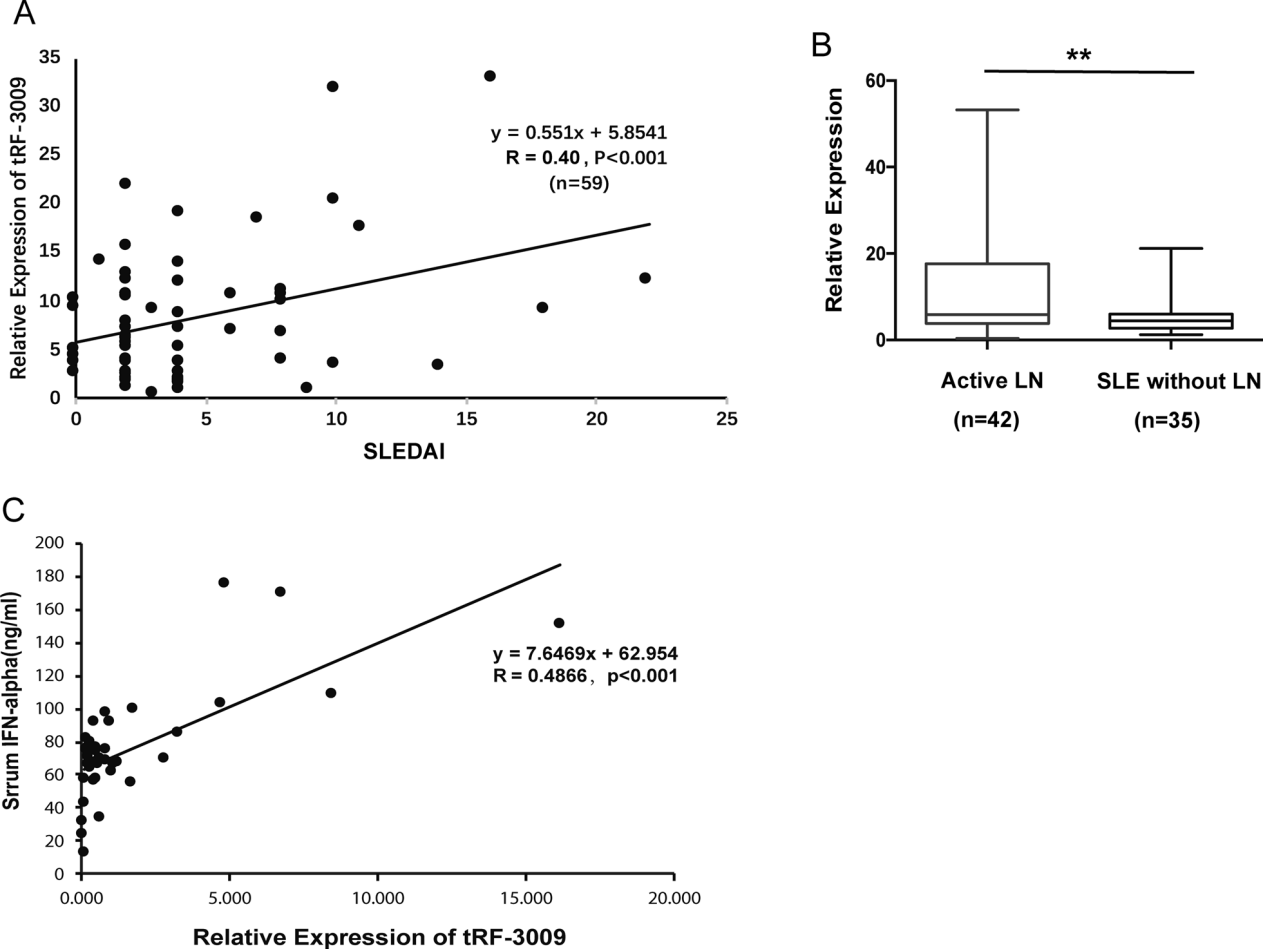
The association between the expression of *tRF-3009* in CD4^+^ T cells and clinical characteristics in systemic lupus erythematosus (SLE) patients. **A** Correlations between the expression of *tRF-3009* and SLEDAI index in SLE patients (n  =  59). **B** The level of *tRF-3009* expression between SLE patients with active nephritis and no nephritis. **P  <  0.01. **C** Correlations between the expression of *tRF-3009* and the level of serum IFN-α in SLE patients (n  =  38)

Based on the difference in the candidate tRFs in the validation cohort, we went on to evaluate the correlations between the expression levels of these *tRFs* in CD4^+^ T cells and clinical phenotype. Among the two candidate up-regulated tRFs, only the expression level of *tRF-3009* in CD4^+^ T cells was positively correlated with SLEDAI (Fig. 2A). Interestingly, *tRF-3009* expression level was significantly higher in patients with active lupus nephritis (LN) than in those without LN (Fig. 2B). Nevertheless, the other three tRFs showed no distinct connection with active SLE (Additional file 1: Figure S1). Among the SLE patients, we found that serum IFN-α levels were positively correlated with *tRF-3009* expression in CD4^+^ T cells (Fig. 2C). Furthermore, we have investigated the relationship between treatments (type and dose) and the levels of tRF-3009. We found the expression level of tRF-3009 showed no significant difference among different type of treatments (hydroxychloroquine and different immunosuppressants; Additional file 2: Figure S2A). Likewise, there was no significant difference in the tRF-3009 expression in different doses of prednisone groups (Additional file 2: Figure S2B). These analyses further suggest that *tRF-3009* has a strong association with SLE in the validation cohort.

### Bioinformatics analysis of tRF-3009 regulated genes

**Fig. 3:**
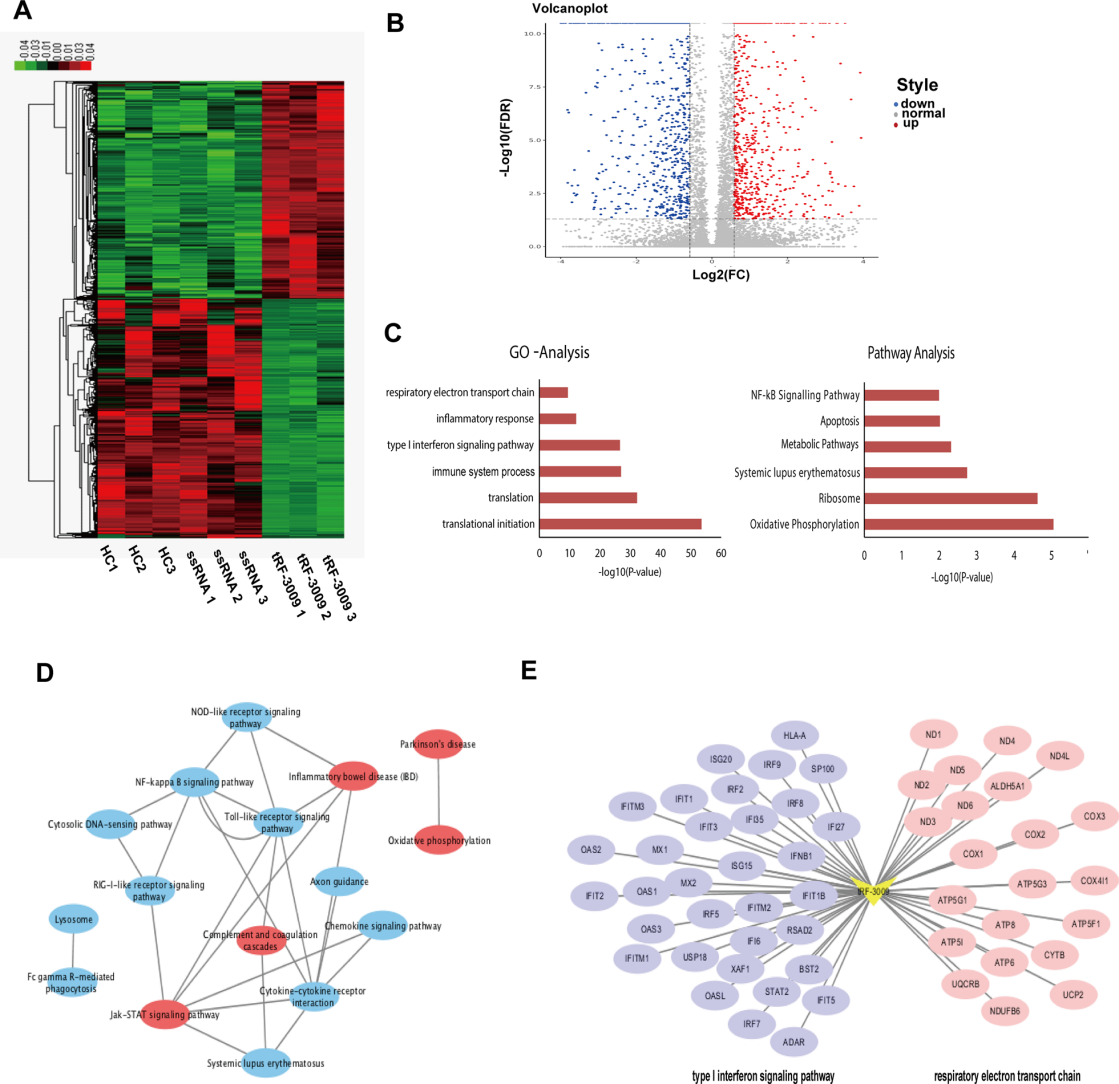
Differentially expressed genes (DEGs) of CD4^+^ T cell transfected with *tRF-3009* and negative control (NC). **A** The Heatmap of DEGs of CD4^+^ T cell from three healthy controls transfected with *tRF-3009* (n  =  3) and NC (n  =  6). The red indicates upregulated genes, and the green indicates downregulated genes. **B** The Volcano plot of DEGs of CD4^+^ T cell transfected with *tRF-3009* (n  =  3) and NC (n  =  6). The red points indicate upregulated genes (n  =  861), and the blue points indicate downregulated genes (n  =  910). **C** Gene ontology (GO) analysis (left panel) and Pathway analysis (right panel) of DEGs. **D** Relation network of KEGG pathways enrichment of DEGs. The red indicates pathways that upregulated DEGs significantly enriched, and the blue indicates pathways that downregulated DEGs significantly enriched. **E** The DEGs related to type I IFN signaling pathway or respiratory electron transport chain. The purple indicates DEGs related to type I IFN signaling pathway, the red indicates DEGs related to respiratory electron transport chain

We used next generation sequencing to explore the molecular function of *tRF-3009* in SLE CD4^+^ T cells. When we compared the *tRF-3009* mimics with the two negative control groups we were able to clearly identify 1771 differentially regulated mRNA transcripts. Of these 861 were upregulated and 910 were downregulated when evaluated using the following criteria: |fold change| ≥  1.5 and FDR  <  0.05 (Fig. 3A, B; Additional file 9: Table S4).

These target genes were then evaluated using GO and pathway analysis. The GO analysis indicated that *tRF-3009* target genes primarily participate in the process of translation, immune and inflammatory responses, the type I IFN response and the respiratory electron transport chain (ETC) (Fig. 3C). Meanwhile, the results of the KEGG analysis showed that the *tRF-3009* target genes were significantly enriched for oxidative phosphorylation, ribosome, ‘systemic lupus erythematosus pathway’ and metabolic pathways (Fig. 3C; Additional file 10: Table S5). To further evaluate the associations between *tRF-3009* target genes and SLE we went on to construct a target-gene-pathway network (Fig. 3D). The results of the Cytoscape analysis also indicate that *tRF-3009* target genes link the type I IFN pathway and oxidative phosphorylation response*.* The visual regulation network was built up between *tRF-3009* and its target genes involved in the type I IFN pathway and respiratory ETC (Fig. 3E). qRT-PCR further validated that over-expression of *tRF-3009* up-regulated genes involved in respiratory ETC, including *ND1* through *ND6* and *ND4L* (Additional file 3: Figure S3).

### IFN-α directly induces tRF-3009 expression and increases OXPHOS in CD4^+^ T cells

**Fig. 4:**
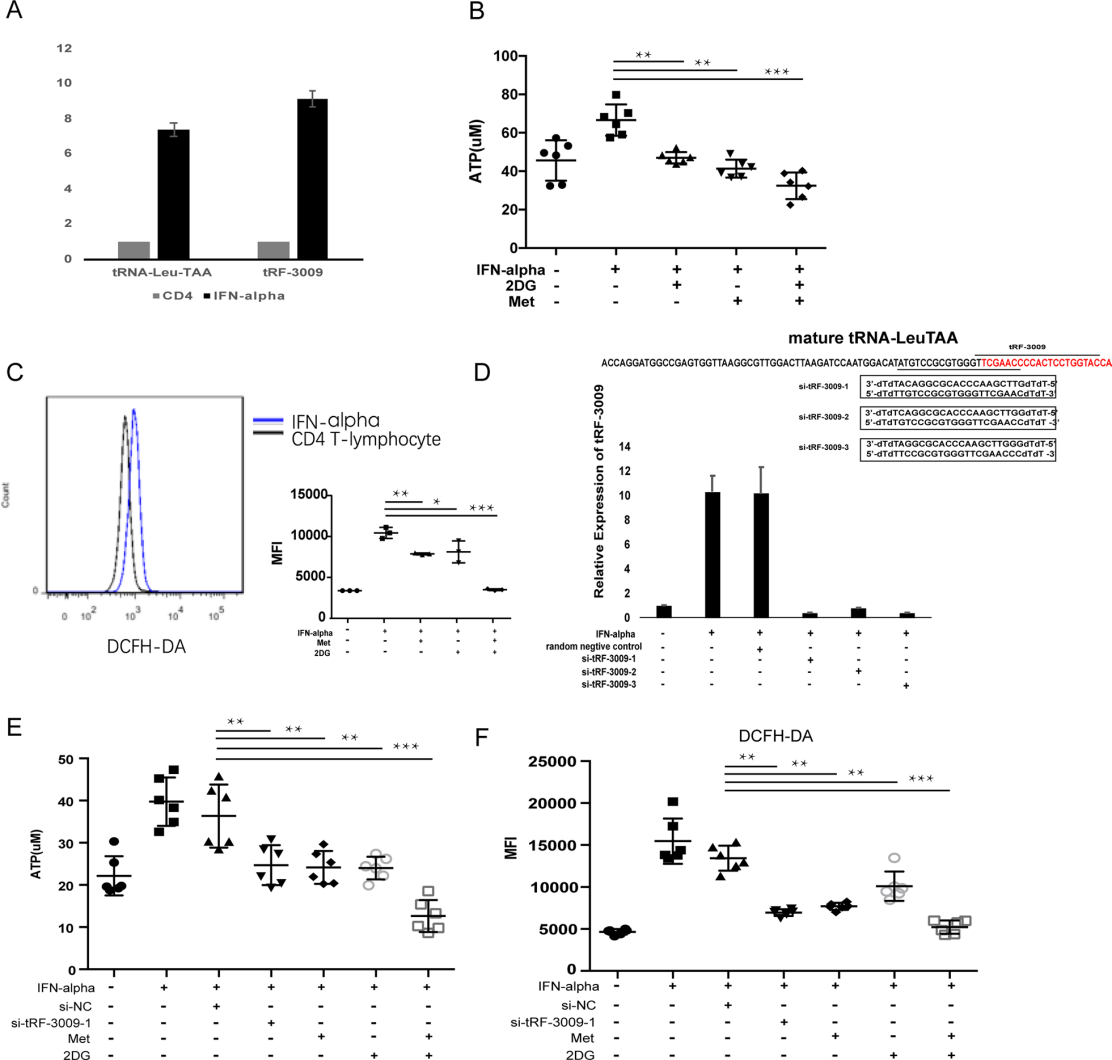
The metabolism change in healthy donor-derived CD4^+^ T cells induced by IFN-α following *tRF-3009* treatment. All experiments show the mean of individual replicates (n  ≥  3) **A** The expression of *tRF-3009* and its precursor tRNA-Leu-TAA in CD4^+^ T cells treated with IFN-α for 24 h by qRT-PCR. Bars show the mean of individual replicates. **B** The change of ATP production in CD4 ^+^ T cells with/without 2-DG or/and metformin after 24-h IFN-α treatment (n  =  6). **C** The change of ROS production in CD4^+^ T cells with/without 2-DG or/and metformin after 24-h IFN-α treatment. **D** The expression of *tRF-3009* in CD4^+^ T cells with IFN-α treatment transfected three siRNA against *tRF-3009* precursor. The sequences of three *si-tRF-3009* and target sites in the *tRF-3009* were shown. **E** The change of ATP production in CD4^+^ T cells induced by IFN-α treatment after transfected *si-tRF-3009-1*(n  =  6). **F** The change of ROS production in CD4^+^ T cells induced by IFN-α treatment after transfected *si-tRF-3009-1* (n  =  6). **P  <  0.01; ***P  <  0.001

Based on our transcriptome data and the results of the bioinformatics analysis, we went on to evaluate the connections between *tRF-3009,* type I IFN and respiratory ETC. First, healthy donor-derived CD4^+^ T cells demonstrated a significant increase in both *tRF-3009* and its precursor tRNA-Leu-TAA expression following IFN-α treatment (Fig. 4A), which suggests IFN-α could directly activate transcription of tRNA-Leu, and may also enhance the fragmentation of tRF-3009 at the same time. And the production of ATP and ROS (products of respiratory ETC) were also increased in these cells after IFN-α treatment (Fig. 4B, C). Then when CD4^+^ T cells where pretreated with metformin or 2-DG and then exposed to IFN-α-induction both the OXPHOS and glycolysis responses were reduced and when these cells were treated with a combination of metformin and 2-DG these effects were completely abolished (Fig. 4B, C). To address whether *tRF-3009* participates in this process, we designed three siRNAs against its tRNA precursor, all of which were able to block the expression of *tRF-3009* when exposed to IFN-α-induction (Fig. 4D). *si-tRF-3009-1* was selected and transfected into healthy donor-derived CD4^+^ T cells and was shown to significantly suppress IFN-α-induced ROS and ATP production (Fig. 4E, F), showing equivalent to the suppressor metformin or 2-DG. Therefore, *tRF-3009* actively participates in the IFN-α-induced respiration metabolism.

### Transfection of tRF-3009 induced oxidative metabolism changes in CD4^+^ T cells

**Fig. 5:**
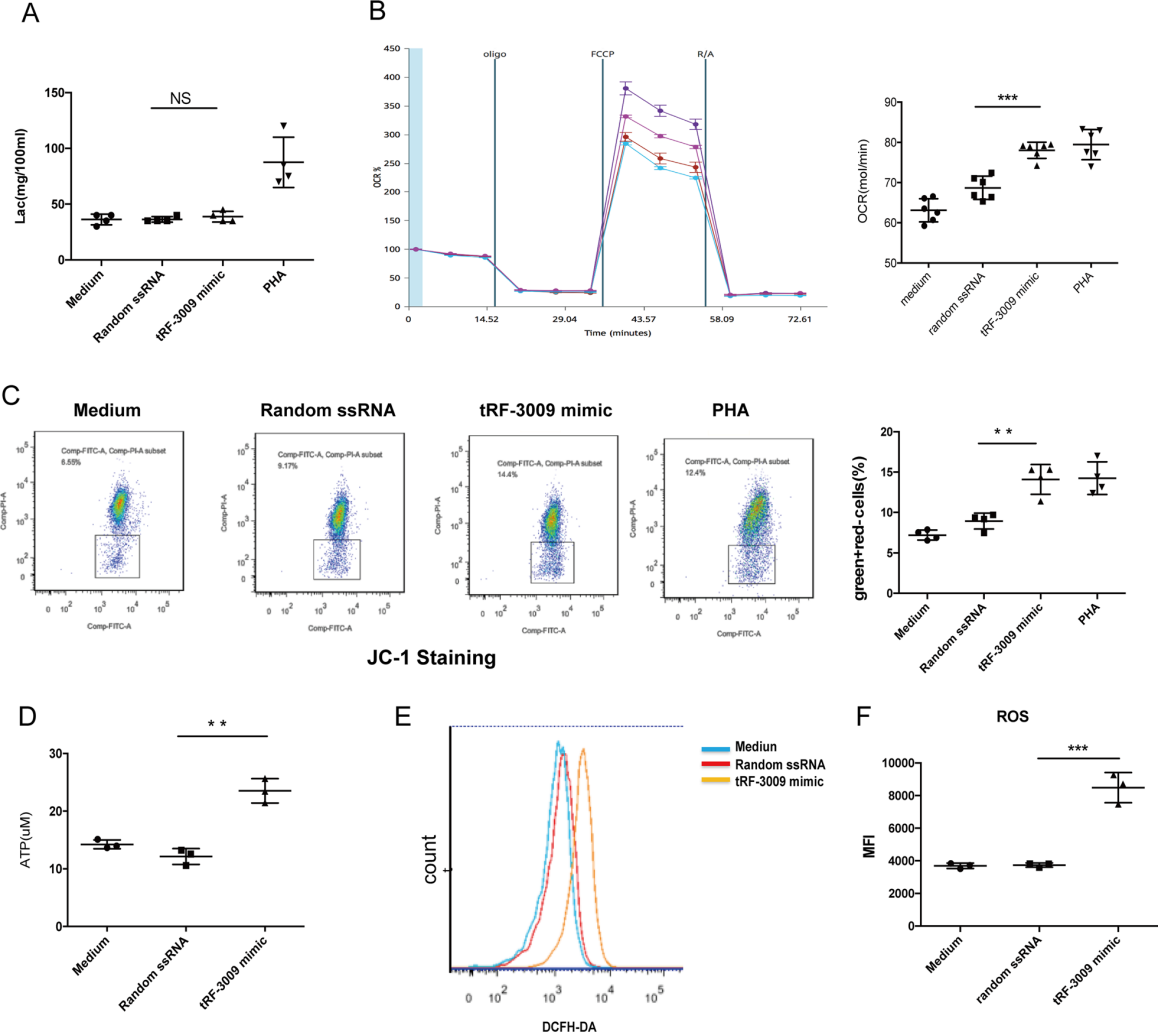
In vitro transfection of *tRF-3009* caused oxidative metabolism changes in healthy donor-derived CD4^+^ T cells. All experiments show the mean of individual replicates (n  ≥  3). **A** The change of lactate level between healthy donor-derived CD4^+^ T cells transfected with *tRF-3009* mimic, negative controls or positive control (n  =  6). The medium and random ssRNA were used as negative controls, and PHA was used as positive control. **B** The change of oxygen consumption rate (OCR) in CD4^+^ T cells transfected *tRF-3009* mimic in vitro (n  =  6)*.* **C** The change of mitochondrial membrane potential (ΔΨm) as estimated by JC-1 in CD4^+^ T cells transfected *tRF-3009* mimic in vitro (n  =  4). The greater the mitochondrial uptake, the greater concentration of JC-1 aggregate forms which have a red fluorescent emission signal, as opposed to the JC-1 monomer that fluoresces green. **D** The change of ATP production in CD4^+^ T cells transfected *tRF-3009* mimic (n  =  3). **E** The change of ROS production as estimated by DCFH-DA dye in CD4^+^ T cells transfected *tRF-3009* mimic (n  =  3). **F** The change of ROS production as estimated by fluorometric analysis in CD4^+^ T cells transfected *tRF-3009* mimic (n  =  3). *NS,* no difference; **P  <  0.01; ***P  <  0.001

To further dissect the exact metabolic pathway affected by *tRF-3009* expression, we went on to evaluate the lactate level of treated CD4^+^ T cells. There were no differences in the lactate levels of these cells (Fig. 5A), suggesting that *tRF-3009* is unlikely to directly modulate glycolysis. As we speculated, mitochondrial oxidative phosphorylation, which is represented by OCR, was significantly increased in CD4^+^ T cells transfected with *tRF-3009* mimic when compared to the negative controls (medium or transfection with ssRNA; Fig. 5B; Additional file 4: Figure S4). The results of the JC-1 staining indicate that the mitochondrial membrane potential in CD4^+^ T cells transfected with *tRF-3009* mimic was also significantly elevated compared with the two negative controls (Fig. 5C). Likewise, ATP and ROS production in CD4^+^ T cells transfected with *tRF-3009* mimic were also enhanced (Fig. 5D–F). These results suggest that *tRF-3009* expression is sufficient to directly regulate OXPHOS.

### The underlying effects of tRF-3009 in the metabolic processes of CD4^+^ T cells

To address the question of how tRF-3009 affects metabolic processes, we performed RNA pull down to explore the immediate “receptor” binding molecule of tRF3009. Here CD4^+^ T cells were evaluated using synthetic biotinylated tRF-3009 to identify interacting proteins. RNA pull down products were subsequently identified by mass spectrometry (LC/MS; Additional file 5: Figure S5A), and the tRF-3009 interacting molecules was shown to be closely related to ATP binding and ion binding/transportation (Additional file 5: Figure S5B). Then the STRING database was used to further explore the potential interaction network between these molecules (Additional file 5: Figure S5C). And the Reactome Pathway enrichment analysis of these interacting proteins showed that these candidate proteins were enriched in the formation of ATP, mitochondrial biogenesis, and respiratory electron transport which are closely related to OXPHOS (Additional file 5: Figure S5D). These data further supported the proposal that tRF-3009 participates in metabolic modulation in CD4^+^ T cell OXPHOS in SLE patients.

## Discussion

The original goal of this study was to study small RNAs in CD4^+^ T cells of SLE patients. Surprisingly, in addition to some miRNAs with well-known function, the small RNA sequencing data also showed a series of tRNA derived fragments(tRFs). This is the first study to identify differentially expressed tRFs in CD4^+^ T cells from SLE patients, which caught our attention. As a new category of small ncRNAs, the potential function of tRFs in the SLE pathogenesis was explored.

In the sequencing data, expression levels and the motif patterns of the tRFs indicated that they are not random products of CD4^+^ T cells. This prompted us to think about the role of tRFs in the pathogenesis of SLE. The correlation between tRNA fragment and clinical indicators of SLE was analyzed, amongst these tRNA fragments*, tRF-3009,* a small fragment (18nt long) processed from the 3′ end of mature tRNA-Leu, was positively correlated with SLEDAI, active lupus nephritis, and the serum IFN-α level, but showed no significant relationship with different treatments in SLE. The correlations with SLEDAI and active lupus nephritis suggested that tRF-3009 may be related to the severity of disease and existence of nephritis. Moreover, the correlation with serum IFN-α level suggested that the expression or function of tRF-3009 may be related to IFN signaling pathway in lupus. Limited by the number of patients, we are unable to determine the potential of this molecule as a disease biomarker this time, which would be our next research goal.

To further explore potential function of tRF-3009 in CD4^+^ T cells of SLE, we performed in vitro transfection of tRF-3009 mimic, to analyze the transcriptome change induced by tRF-3009 over expression. Transcriptome and bioinformatics analysis suggested that multiple SLE-related pathways were activated in response to this tRF. More specifically, the results of the GO and KEGG analyses identified the potential effects of *tRF-3009* expression on type I IFN signaling and OXPHOS metabolism in CD4^+^ T cells. As reported previously [29], mitochondrial hyperpolarization has been by several potential sensors in lupus T cells, along with increased mitochondrial ROS production and diminished ATP synthesis. Therefore, to confirm tRF-3009 may participate in OXPHOS, we determine the change of mitochondrial membrane potential, ROS and ATP production caused by tRF-3009. Our data further demonstrated that IFN-α induces CD4^+^ T cell OXPHOS in a *tRF-3009-*dependent manner and that *tRF-3009* knockdown suppressed IFN-α-induced ROS production and ATP biogenesis in vitro. This result reflected *tRF-3009* knockdown and metformin or 2-DG have similar moderate effect in modulating OXPHOS and glycolysis responses. Furthermore, *tRF-3009* overexpression upregulates OXPHOS but not glycolysis in CD4^+^ T cells, indicating that this small RNA may serve as a metabolic modulator downstream of the type I IFN pathway, which in turn could enhance OXPHOS in CD4^+^ T cells of SLE.

The serum IFN-α level of SLE patients showed positive correlation with tRF-3009 expression, IFN-α also could promote *tRF-3009* and precursor of *tRF-3009* expression in vitro, indicating a transcriptional regulation of IFN-α in CD4^+^ T cells. So here we suspected that IFN-α may be an important character in tRF-3009 expression and function. Also, our findings raise a ‘chicken or egg’ scenario for the type I IFN response and OXPHOS metabolism. A previous study demonstrated that mitochondrial oxidative stress is a key source of type I IFN production in SLE, evident by enhanced NETosis and oxidized mitochondrial DNA, strongly stimulating type I IFN signaling [30]. As for the natural type I IFN-producing cells, that is, plasmacytoid dendritic cells (pDC), it has been reported that IFN-α can upregulate pDC fatty acid oxidation and OXPHOS via an autocrine type I IFN receptor-dependent pathway [31]. Moreover, our data suggests that the metabolic OXPHOS in CD4^+^ T cells, which have type I IFN receptors on their cell surface, may be regulated by type I IFN in the context of SLE. Currently available data appears to give us more of a snapshot of disease progression reflecting different aspects of the complex and dynamic interactions between IFN responses and cellular metabolism in different immune cells. This means that while the data may seem contradictory it is more likely that there is a complex feedback network regulating IFN signaling and mitochondrial oxidative stress in SLE. Along with the clinical progress of repurposing metabolic modulators, such as metformin, in the management of SLE [8, 32, 33], our data may offer additional insight into the immunometabolism of SLE.

Furthermore, attempts to predict the target genes of *tRF-3009* (3′UTR region and promoter region) using miRNA prediction databases were a failure due to the lack of intersection between predicted target genes and transcriptome data (Additional files 9, 11, 12: Tables S4, S6, S7). The prediction method for miRNA is probably not applicable for tRFs, which might indicate that tRFs function in a different way. Additionally, we performed RNP pull down using *tRF-3009* and analyzed the resulting immunoprecipitation products to identify possible targets. With further bioinformatics analyses, the results suggested that these potential interaction partners were enriched in ATP production, mitochondrial biogenesis, and respiratory electron transport, which are closely related to OXPHOS. These results were preliminary without independent validation, and we are still in the early phases of developing a roadmap to evaluate the precise mechanisms of *tRF-3009* mediated metabolic regulation.

Still, the major limitation of our study is the lack of a mechanistic explanation for the OXPHOS modulating effect of *tRF-3009*. For example, it is still questionable how ATP production is affected by tRF-3009. Lupus PBL has been previously demonstrated to exhibit increased O_2_ consumption through mitochondrial ETC complex I [34]. However, in the current study, the Seahorse assay could not permit functional analysis of individual ETC complexes. And we failed to determine increased ATP production originate from complex I or distributed across several complexes in SLE over healthy control or RA T cells. Another limitation of our study is the lack of biomarker-scale sample size and cross-sectional data, which can’t speculate the potential and development of biomarkers. As reported previously, RA subjects T cells also display rebalancing glucose utilization and restoring oxidant signaling [35]. In view of the metabolic regulatory links of the tRF-3009, the conclusion should be further validated in RA patients. The clinical significance of *tRF-300*9 in SLE is still undetermined and a larger prospective longitudinal study is pending.

## Conclusion

In summary, we demonstrate that *tRF-3009* participate in metabolic modulation in IFN-α-induced CD4^+^ T cell OXPHOS in SLE patients. This means that *tRF-3009* may serve as a potential therapeutic target and may help to advance our understanding of CD4^+^ T cell pathogenesis in SLE.

## Supplementary Information

**Additional file 1: Figure S1.** The association between the expressions of *tRF-3021*, *tRF-1001*, and *tRF-1035* in CD4^+^ T cells and clinical characteristics in systemic lupus erythematosus (SLE) patients.

**Additional file 2: Figure S2.** The relationship between treatments and the levels of tRF-3009 in SLE patients. **A** The expression level of tRF-3009 among different type of treatments (hydroxychloroquine and different immunosuppressants). **B** The tRF-3009 expression in different doses of prednisone groups.

**Additional file 3: Figure S3.** Validation of tRF-3009 target genes expressions in respiratory electron transport chain. After transfection of tRF-3009, the change of the candidate target genes expression in CD4^+^ T cells. Data are presented as 2^−ΔCt^ relative to β-actin expression. Bars show the mean of individual replicates (n = 3).

**Additional file 4: Figure S4.** In vitro transfection of tRF-3009 induced metabolism changes in CD4^+^ T cells using Seahorse assay. Proton leak (**A**) and ATP production (**B**) in CD4^+^ T cells transfected tRF-3009 mimic or random ssRNA. O_2_ consumption (**C**) and Energy Map (**D**) of CD4^+^ T cells transfected tRF-3009 mimic or random ssRNA.

**Additional file 5: Figure S5.** The potential mechanistic role of tRF-3009 in CD4^+^ T cells in terms of regulating OXPHOS. **A** The immunoprecipitation products from RNA pull down using tRF-3009 were shown by mass spectrometry (LC/MS). **B** The potential “receptor” binding molecule of tRF-3009. **C** Protein and protein interaction (PPI) network of the potential “receptor” binding molecule of tRF-3009. **D** Rectome Pathway analysis of the potential “receptor” binding molecule of tRF-3009. The size of the spots represents the number of the potential molecule of tRF-3009, and the color of the spots represents the P value.

**Additional file 6: Table S1.** Clinical charateristics of SLE patients, disease controls and healthy controls.

**Additional file 7: Table S2.** Primers used for qRT-PCR.

**Additional file 8: Table S3.** Differentially expressed tRFs in CD4^+^ T cells of SLE patients and healthy controls (Has).

**Additional file 9: Table S4.** Differentially expressed genes between transfection of tRF-3009 and NC groups.

**Additional file 10: Table S5.** The KEGG analysis of differentially expressed genes in the ‘systemic lupus erythematosus pathway’.

**Additional file 11: Table S6.** Predicted target 3′UTR regions of tRF-3009.

**Additional file 12: Table S7.** Predicted target promoter regions of tRF-3009.

## Data Availability

All data generated or analyzed in this study are included in this article and its Supplementary materials.
