# Supplementary figures and images for: tRNA derived fragment (tRF)-3009 participates in modulation of IFN-α-induced CD4+ T cell oxidative phosphorylation in lupus patients
Source: J Transl Med. 2021 Jul 13;19:305. doi: 10.1186/s12967-021-02967-3 (PMC8278670; doi:10.1186/s12967-021-02967-3)

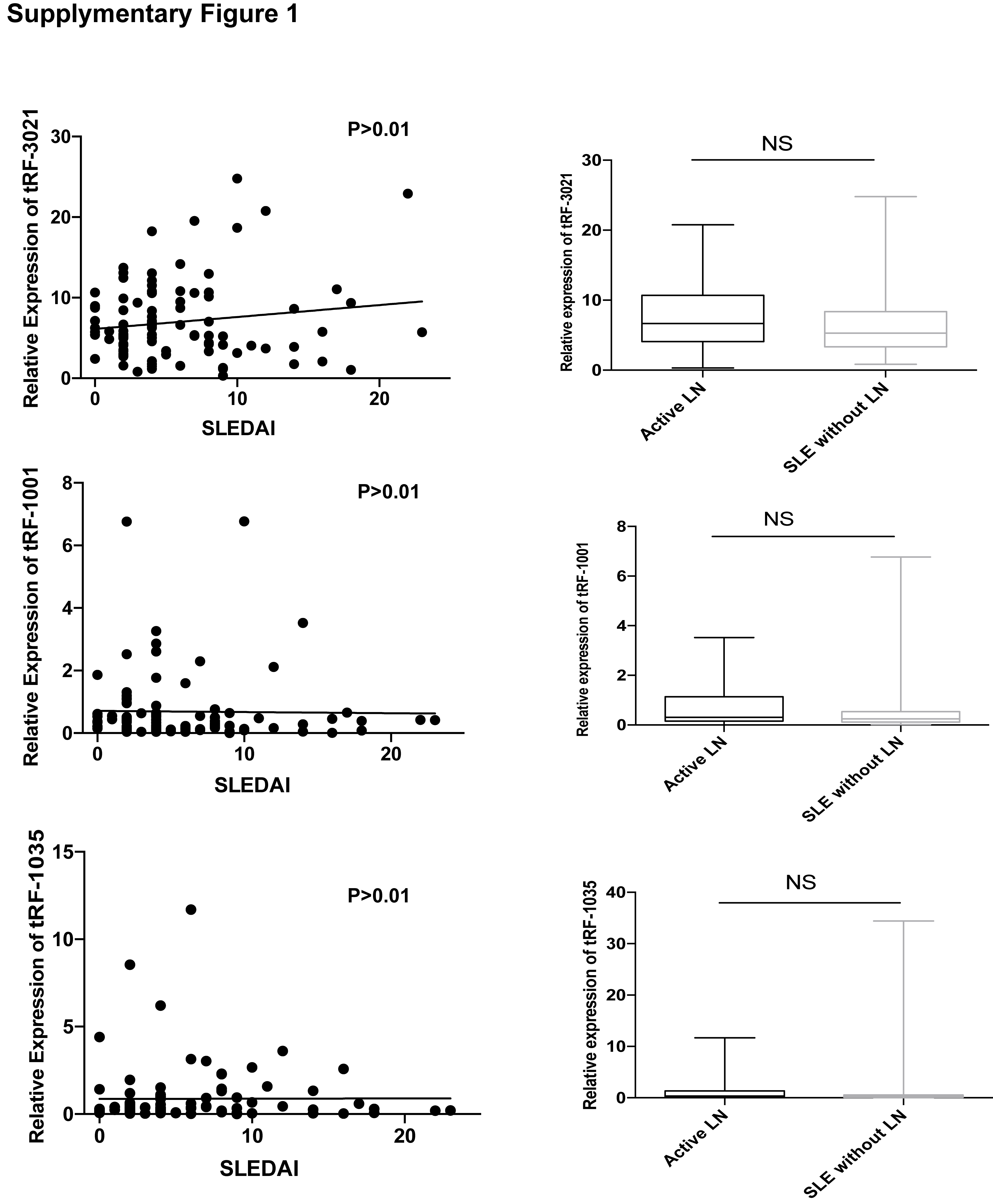

Supplement: Supplementary file 1 — Additional file 1: Figure S1. The association between the expressions of tRF-3021, tRF-1001, and tRF-1035 in CD4+ T cells and clinical characteristics in systemic lupus erythematosus (SLE) patients. [file 12967_2021_2967_MOESM1_ESM.tif]

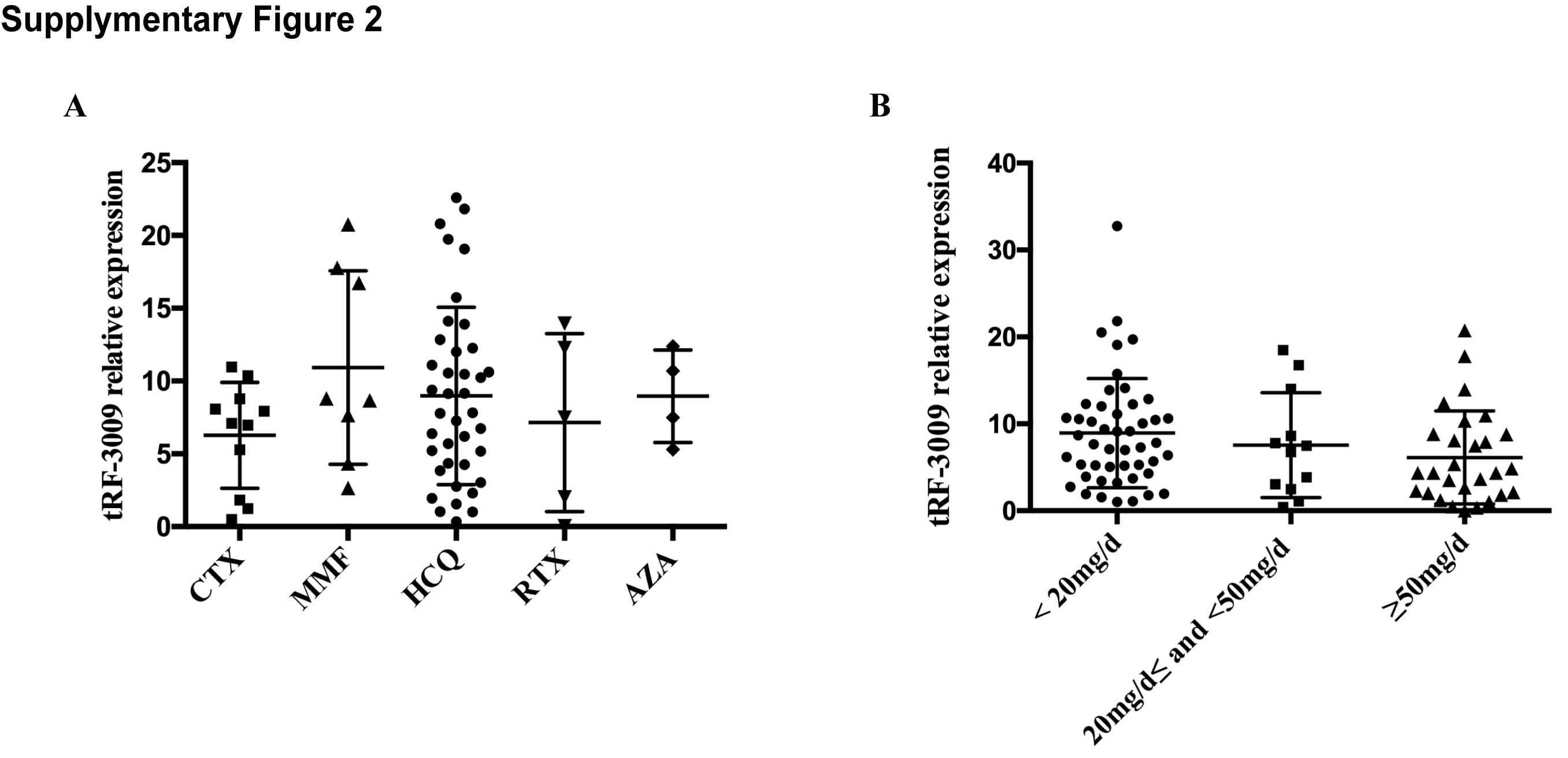

Supplement: Supplementary file 2 — Additional file 2: Figure S2. The relationship between treatments and the levels of tRF-3009 in SLE patients. A The expression level of tRF-3009 among different type of treatments (hydroxychloroquine and different immunosuppressants). B The tRF-3009 expression in different doses of prednisone groups. [file 12967_2021_2967_MOESM2_ESM.tif]

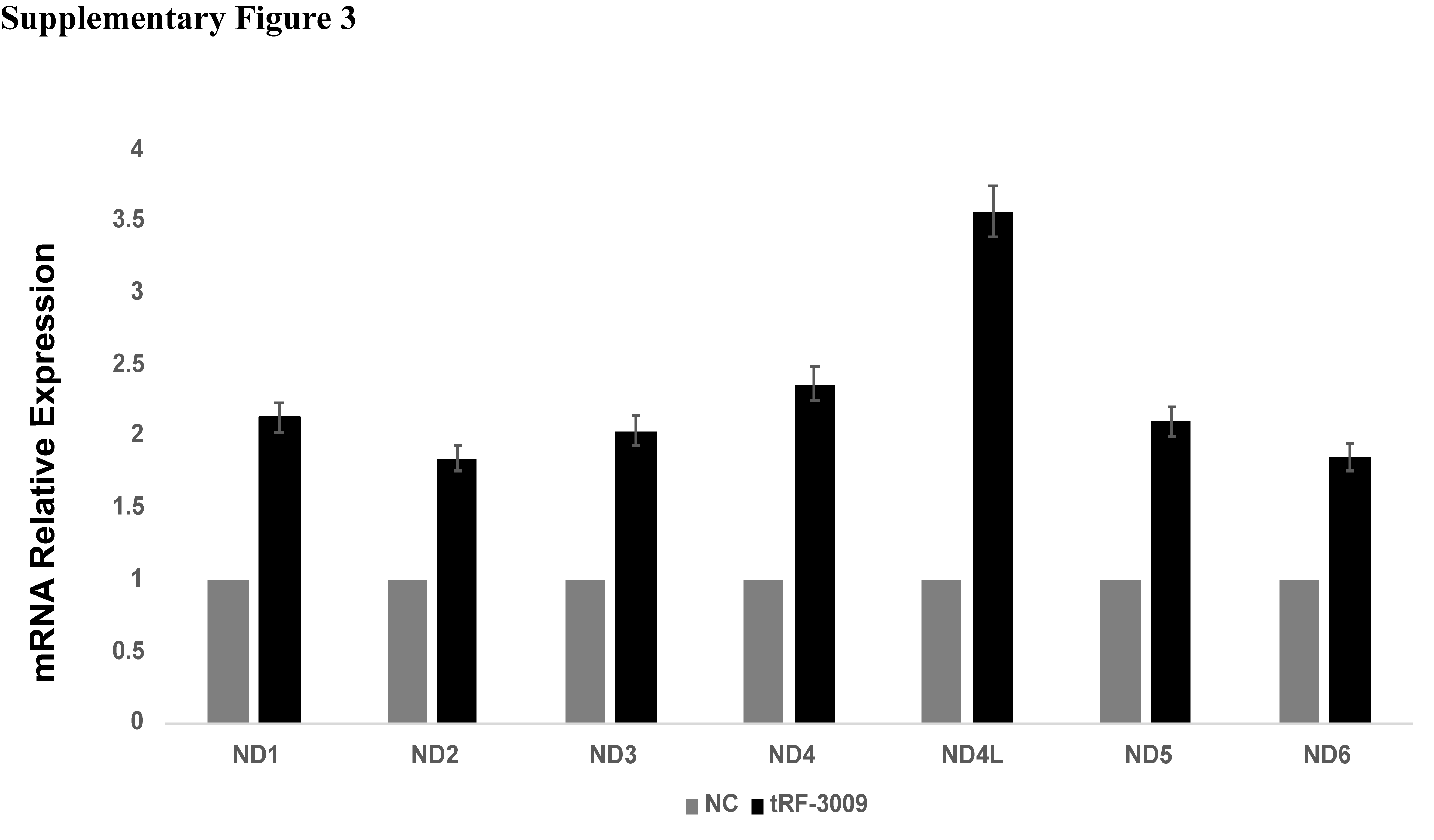

Supplement: Supplementary file 3 — Additional file 3: Figure S3. Validation of tRF-3009 target genes expressions in respiratory electron transport chain. After transfection of tRF-3009, the change of the candidate target genes expression in CD4+ T cells. Data are presented as 2−ΔCt relative to β-actin expression. Bars show the mean of individual replicates (n = 3). [file 12967_2021_2967_MOESM3_ESM.tif]

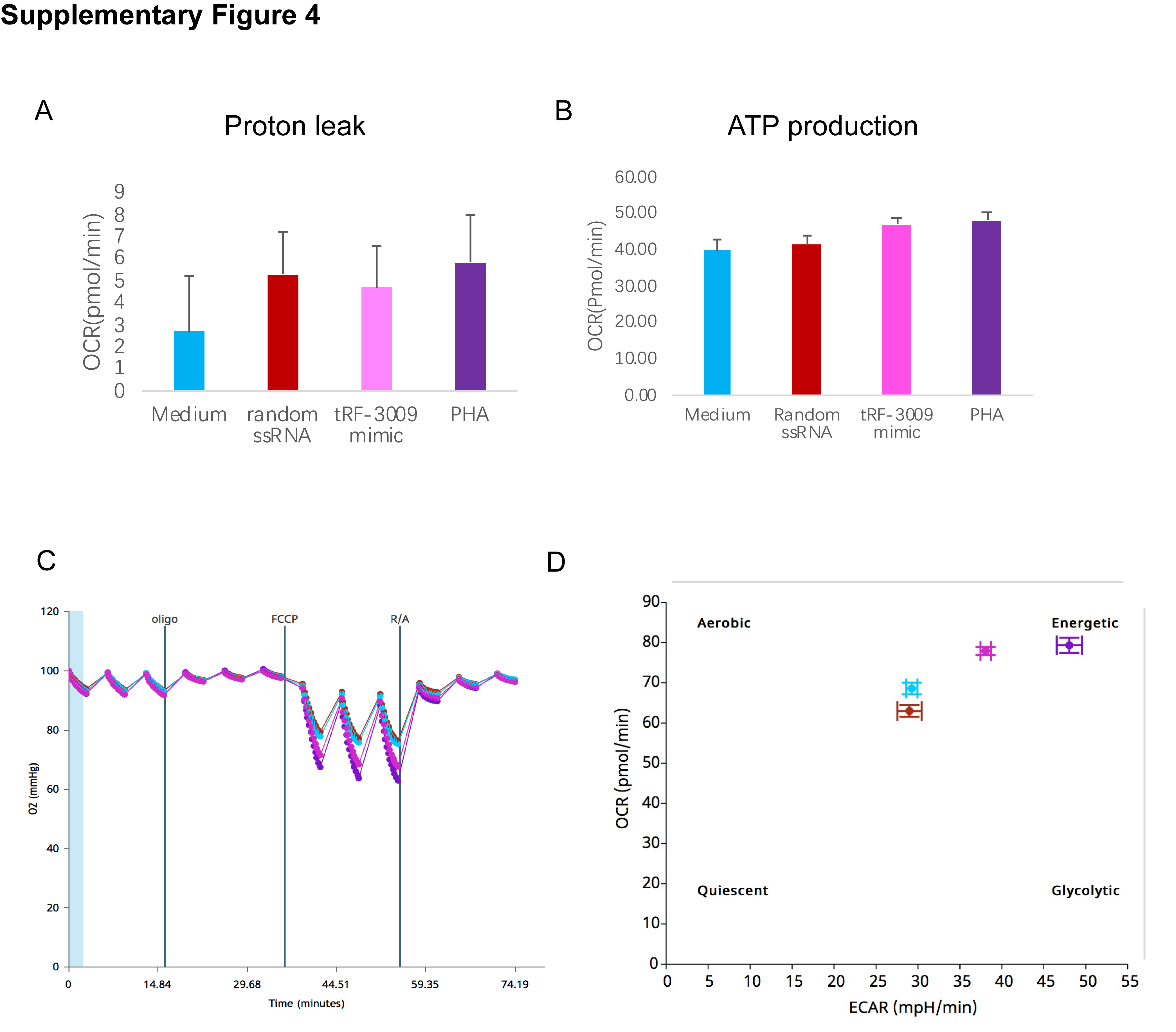

Supplement: Supplementary file 4 — Additional file 4: Figure S4. In vitro transfection of tRF-3009 induced metabolism changes in CD4+ T cells using Seahorse assay. Proton leak (A) and ATP production (B) in CD4+ T cells transfected tRF-3009 mimic or random ssRNA. O2 consumption (C) and Energy Map (D) of CD4+ T cells transfected tRF-3009 mimic or random ssRNA. [file 12967_2021_2967_MOESM4_ESM.tif]

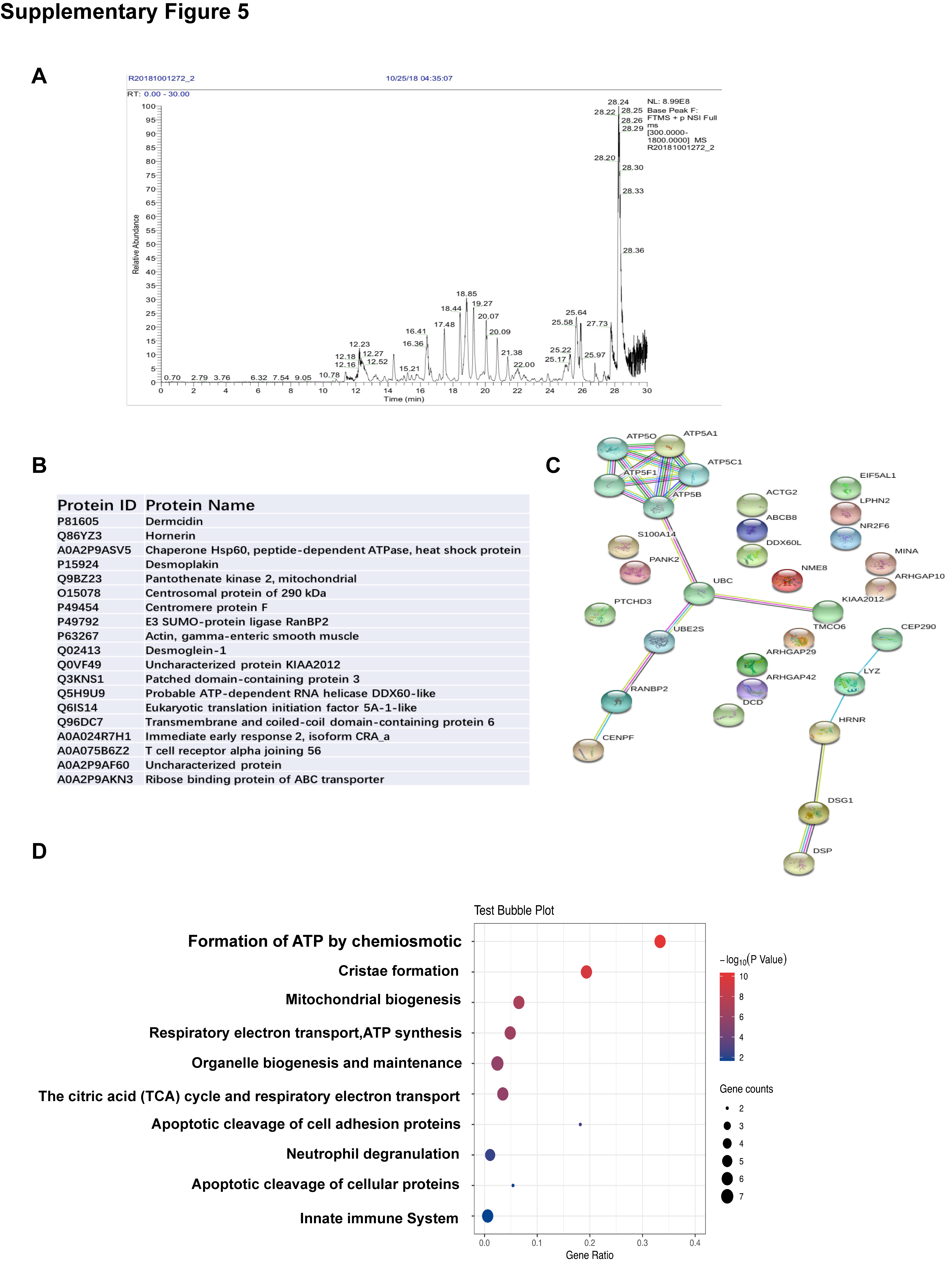

Supplement: Supplementary file 5 — Additional file 5: Figure S5. The potential mechanistic role of tRF-3009 in CD4+ T cells in terms of regulating OXPHOS. A The immunoprecipitation products from RNA pull down using tRF-3009 were shown by mass spectrometry (LC/MS). B The potential “receptor” binding molecule of tRF-3009. C Protein and protein interaction (PPI) network of the potential “receptor” binding molecule of tRF-3009. D Rectome Pathway analysis of the potential “receptor” binding molecule of tRF-3009. The size of the spots represents the number of the potential molecule of tRF-3009, and the color of the spots represents the P value. [file 12967_2021_2967_MOESM5_ESM.tif]
